# Oestrogen receptor negative breast cancers exhibit high cytokine content

**DOI:** 10.1186/bcr1648

**Published:** 2007-01-29

**Authors:** Carine Chavey, Frédéric Bibeau, Sophie Gourgou-Bourgade, Sandrine Burlinchon, Florence Boissière, Daniel Laune, Sylvie Roques, Gwendal Lazennec

**Affiliations:** 1 INSERM, U844, Site Saint Eloi, Bâtiment INM, University of Montpellier I, rue Augustin Fliche, Montpellier, F-34091, France; Montpellier, F-34090, France; 2 CRLC Val d'Aurelle, Pathology Department, rue des Apothicaires, Montpellier, F-34298, France; 3 CRLC Val d'Aurelle, Biostatistics Unit, rue des Apothicaires, Montpellier, F-34298, France; 4 CNRS, UMR 5160, Centre de Pharmacologie et Biotechnologie pour la Sante, Faculte de Pharmacie, avenue Charles Flahault, Montpellier, F-34093, France

## Abstract

**Introduction:**

An emerging hypothesis suggests that cytokines could play an important role in cancer as potential modulators of angiogenesis and leucocyte infiltration.

**Methods:**

A novel multiplexed flow cytometry technology was used to measure the expression of 17 cytokines (IL-1β, IL-2, IL-4, IL-5, IL-6, IL-7, IL-8, IL-10, IL-12 [p70], IL-13, IL-17, granulocyte colony-stimulating factor [CSF], granulocyte-macrophage CSF, IFN-γ, monocyte chemoattractant protein [MCP]-1, macrophage inflammatory protein [MIP]-1β, tumour necrosis factor [TNF]-α) at the protein level in 105 breast carcinoma. B lymphocyte, T lymphocyte and macrophage levels were determined by immunohistochemistry.

**Results:**

Fourteen of the 17 cytokines were expressed in breast carcinoma, whereas only nine cytokines could be detected in normal breast. Most cytokines were more abundant in breast carcinoma than in normal breast, with IL-6, IL-8, granulocyte CSF, IFN-γ, MCP-1 and MIP-1β being very abundant. IL-2, IL-6, IL-8, IL-10, IFN-γ, MCP-1, MIP-1β and TNF-α, and to a lesser extent IL-1β and IL-13 exhibited levels of expression that were inversely correlated to oestrogen receptor and progesterone receptor status. Most cytokines were not correlated with age at cancer diagnosis, tumour size, histological type, or lymph node status. However, IL-1β, IL-6, IL-8, IL-10, IL-12, MCP-1 and MIP-1β were more abundant in high-grade tumours than in low-grade tumours. In addition, IL-8 and MIP-1β were expressed to a greater degree in HER2-positive than in HER2-negative patients. The expression of most of the studied cytokines was correlated to levels of activator protein-1, which is known to regulate numerous cytokines. Overexpression of MCP-1 and MIP-1β were linked to B lymphocyte, T lymphocyte and macrophage infiltration, whereas high levels of IL-8 were correlated with high macrophage content in tumour. Moreover, IL-8 positive tumours exhibited increased vascularization.

**Conclusion:**

We found that multiple cytokines were overexpressed in oestrogen receptor negative breast carcinoma, and that the three major cytokines – MCP-1, MIP-1β and IL-8 – were correlated with inflammatory cell component, which could account for the aggressiveness of these tumours.

## Introduction

Breast cancer represents the leading cause of cancer death among women in developed countries [1]. Among the various prognostic factors, lack of oestrogen receptor (ER) has consistently been associated with poorer prognosis [2]. Most human breast cancers express ER-α, and the presence of this receptor is generally considered an indication of hormone dependence [3]. In addition to ER-α, cytokines are now emerging as factors that are potentially involved in breast carcinogenesis [4,5]. Cytokines constitute a diverse group of proteins that include haematopoietic growth factors, interferons, lymphokines and chemokines [6].

Cytokines are produced by many cell populations, but the predominant suppliers are T-helper (Th) cells and macrophages. Th cells have two important functions: to stimulate cellular immunity and inflammation, and to stimulate B cells to produce antibody. Two functionally distinct subsets of Th cells (Th1 and Th2) secrete cytokines that promote these different activities. Th1 cells produce IL-2 and IFN-γ, which activate cytotoxic lymphocytes and macrophages to stimulate cellular immunity and inflammation [7]. Th2 cells secrete IL-4 and IL-5, which stimulate antibody production by B cells.

It has become evident that cancer tissues also produce cytokines [4,8]. Numerous studies have analyzed the expression of diverse cytokines independently in breast cancer, but only a few have examined the profile of a group of cytokines at the tumour site. In the present study we screened 17 members of the cytokine family (IL-1β, IL-2, IL-4, IL-5, IL-6, IL-7, IL-8, IL-10, IL-12 [p70], IL-13, IL-17, granulocyte colony-stimulating factor [G-CSF], granulocyte-macrophage colony-stimulating factor [GM-CSF], IFN-γ, monocyte chemoattractant protein [MCP]-1, macrophage inflammatory protein [MIP]-1β and tumour necrosis factor [TNF]-α) in breast cancer tissues. We found that most cytokines were overexpressed in breast cancer compared with normal tissues. IL-2, IL-6, IL-8, IL-10, INF-γ, MCP-1, MIP-1β, TNF-α, IL-1β and IL-13 were inversely correlated with ER and progesterone receptor (PR) status. Most of the cytokines were not correlated with patient age, tumour size, histological type, or lymph node status. IL-1β, IL-6, IL-8, IL-10, IL-12, MCP-1 and MIP-1β were abundant in high-grade tumours. Moreover, IL-8 and MIP-1β were linked to HER2 expression. In addition, IL-8 positive tumours exhibited greater vascularization. Robust expression of IL-8, MCP-1 and MIP-1β was correlated with a strong inflammatory cell component.

## Materials and methods

### Patients and samples

Breast tumour surgical specimens were selected from the files of the Pathology Department (CRLC Val d'Aurelle) tumour bank. Breast biopsies were from surgical waste; they were not formal samples taken for diagnosis and they did not undergo genetic evaluation. The samples were selected by a pathologist from fresh surgical specimens immediately after resection. For each sample, a twin sample from the same tissue was taken at the same time, embedded in paraffin and evaluated to ensure that the tissue was indeed tumourous. When possible, a sample of normal breast tissue in the vicinity of the tumour was also taken. All of the patients had primary unilateral, nonmetastatic breast carcinoma, and complete clinical, histological and biological information was available for all of them. The clinical and pathological characteristics of the patients are given in Additional file 1. The tumours included invasive carcinoma, mainly ductal and lobular, and other rare subtypes (mixed ductal and lobular carcinoma, medullary carcinoma and apocrine carcinoma). The tumours were graded according to the modified Nottingham SBR system [9] and categorized according to the sixth edition of the AJCC (American Joint Committee on Cancer) Cancer Staging Manual for pTNM staging. ER-α, progesterone receptor (PR) and HER2 status were determined at the protein level by immunohistochemistry. When equivocal, HER2 results were confirmed by fluorescence *in situ *hybridization.

### Immunohistochemistry and fluorescence *in situ *hybridization analysis

Representative tissue sections from cases of invasive breast carcinoma were fixed in 10% formalin or in alcohol-formalin-acetic acid and embedded in paraffin. Sections from each case were deparaffinized, rehydrated and subsequently subjected to heat induced antigen retrieval by immersing them, depending on the antibody, either in a water bath with a sodium citrate buffer (pH 6) or an EDTA buffer (pH 8). Immunohistochemistry was performed using a Dako autostainer (Dako, Glostrup, Denmark). Then, the sections were incubated with the following primary antibodies: ER-α (clone 6F11, monoclonal; Novocastra, Newcastle-Upon-Tyne, UK), PR (clone PgR636, monoclonal; DakoCytomation, Glostrup, Denmark) and HER 2 (A0485, polyclonal; DakoCytomation). They were respectively used at 1:50, 1:100 and 1:1500 dilutions with an incubation time of 30 min. Antibody was localized using the LSAB^® ^2 Detection System (Dako). Diaminobenzidine (Dako) was used as the chromogen and the sections were lightly counterstained with haematoxylin. ER and PR expression was considered positive when at least 10% of invasive tumoural cells exhibited nuclear staining, regardless of intensity. For HER2, the immunohistochemical score was assigned according to the Herceptest^® ^scoring system (0 = absence of membranous staining or fewer than 10% positive cells; 1+ = more than 10% stained cells with weak and incomplete membranous staining; 2+ = more than 10% stained cells with weak or moderate complete membranous staining; and 3+ = more than 10% stained cells with strong and complete membranous staining). A case was considered to be HER2 overexpressing if it scored 3+. A case scoring 2+ was considered to be HER2 overexpressing only if fluorescence *in situ *hybridization analysis using the PathVysion HER-2 Probe Kit (Vysis, Downer's Grove, IL, USA) revealed HER2 gene amplification.

The inflammatory cell component was evaluated in a subset of 10 tumours exhibiting high levels of IL-8 and 10 tumours with low IL-8 content using CD3 (T-lymphocyte lineage), CD20 (B-lymphocyte lineage) and CD68 (macrophages) antibodies. The intensity of each pattern of inflammatory infiltrate was graded as absent (0), minimal (1), moderate (2), or marked (3). The staining result for each antibody was scored by two investigators who were blinded as to the IL-8 status of the patients. Vessels were assessed within the invasive carcinoma in CD31-stained sections. The most vascular areas were identified by examination at low power (× 40 magnification). The number of clusters or single cells stained for CD31 was recorded from the three most vascular × 400 fields (0.18 mm^2 ^each) and the vascular density (number of vessels/mm^2^) was calculated.

### Protein extract preparation

Biopsies were first crushed in liquid nitrogen. The powder was then resuspended in TEG (10 mmol/l Tris-HCl [pH 7.4], 1.5 mmol/l EDTA and 10% glycerol) containing protease inhibitors (5 μg/ml aprotinin, leupeptin and pepstatin A, and 0.1 mmol/l phenylmethylsulfonyl fluoride). The mixture was then sonicated and the cellular debris was pelleted by centrifugation at 13,000 *g *for 20 minutes at 4°C in microfuge tubes.

### Cytokine multiplexed bioplex assay

A panel of 17 cytokines (IL-1β, IL-2, IL-4, IL-5, IL-6, IL-7, IL-8, IL-10, IL-12 [p70], IL-13, IL-17, G-CSF, GM-CSF, IFN-γ, MCP-1, MIP-1β, TNF-α) was measured in duplicate using the bioplex cytokine multiplexed assay (BioRad, Hercules, CA, USA), in accordance with the manufacturer's instructions (Additional file 2). This novel multiplexed, particle-based, flow cytometric assay uses anticytokine monoclonal antibodies linked to microspheres incorporating distinct proportions of two fluorescent dyes. Five hundred micrograms of total protein extracts of breast biopsies was used to measure cytokine concentration. For each cytokine, eight standards ranging from 2 to 32,000 pg/ml were used, and the minimum detectable dose was 10 pg/ml. The assay was validated by comparing the results obtained by bioplex measure of IL-8 with a conventional enzyme-linked immunosorbent assay measure of IL-8 (Additional file 3).

### Gel shift assays

Gel shift assays were performed as described previously [10]. Briefly, 30,000 count per minute of the [^32^P]-labelled double-strand oligonucleotides were combined with 1 μg poly(dI-dC) and 5 μg total cell extract. The reaction buffer contained 20 mmol/l Hepes (pH 7.9), 1 mmol/l DTT, 50 mmol/l KCl, 10% glycerol and 2.5 mmol/l MgCl_2_. Protein-DNA complexes were separated from the free probe by nondenaturating gel electrophoresis with 4% polyacrylamide gels in 0.5 × TBE. We used the activator protein (AP)-1 consensus oligonucleotide: 3'-CGCTTGATGAGTCAGCCGGAA-5'. The levels of shifted complexes were detected and quantified by counting using a Fujix-Bas 1000 (Fuji Photo-Film, Tokyo, Japan).

### Statistical analysis

Data were summarized by frequency for categorical variables and by median and range for continuous variables. Correlation between variables (cytokines) was evaluated using the Spearman correlation coefficient after log transformation. Associations between categorical variables were examined using χ^2 ^analyses.

The median levels of expression of analyzed interleukins were compared using Kruskal-Wallis test according to clinical data (age, ER, PR, SBR status, pT and pN status) or analysis of variance if possible.

For all statistical analysis, *P *< 0.05 was considered statistically significant.

## Results

### Breast tumours overexpress cytokines

A total of 105 samples of primary unilateral, nonmetastatic breast carcinomas (Additional file 1) and 13 healthy breast biopsies were analyzed for cytokine expression at the protein level (Table 1). This was made possible by the use of a novel technology that couples enzyme-linked immunosorbent assay to clourescence-activated cell sorting analysis, which allows detection of multiple proteins in a single well.

We observed that healthy breast samples expressed very low levels of cytokines except for IFN-γ and MIP-1β (Table 1). In contrast, breast tumours exhibited high levels of IL-6, G-CSF and IFN-γ, and extremely high levels of IL-8, MCP-1 and MIP-1β. The cytokines IL-5, IL-17 and GM-CSF were not detected in either normal or tumour sample, whereas IL-1β, IL-2, IL-4, G-CSF and IL-10 were only detected in breast carcinoma. IL-6, IL-8, IL-12, IL-13, IFN-γ, MCP-1, MIP-1β and TNF-α were significantly more abundant in carcinoma than in normal breast. The greatest differences in expression between normal breast tissue and carcinoma occurred in IL-8, MCP-1 and MIP-1β, which were 60-fold more abundant in carcinoma than in healthy breast, followed by IL-6, which was about 14-fold overexpressed in carcinoma. In summary, we found that breast tumours express higher levels of multiple cytokines than do normal tissues.

### Correlation between cytokines and ER and PR

We next evaluated whether there was any correlation between cytokine expression and ER levels (Table 2). We observed that IL-4 and G-CSF were not correlated with ER status. On the other hand, IL-2, IL-6, IL-8, IL-10, IFN-γ, MCP-1, MIP-1β, TNF-α and, to a lesser extent, IL-1β and IL-13 were significantly overexpressed in ER-negative tumours compared with ER-positive ones, with the greatest differences observed for IL-8 and MCP-1.

We performed the same type of analysis to identify whether there was any correlation between cytokine levels and PR expression (Table 2). IL-2 and IL-13 were not correlated with PR status, whereas IL-1β, IL-6, IL-8, IL-10, IFN-γ, MCP-1, MIP-1β, TNF-α and, to a lesser extent, IL-4, IL-12 and G-CSF were more abundant in PR-negative tumours than in PR-positive ones. The greatest differences were obtained for IL-8, IL-6 and MCP-1.

The fact that some cytokines were correlated with either ER or PR led us to analyze the distribution of all cytokines by combining both factors (Additional file 4). Three categories were defined, corresponding to true ER negative (ER^-^/PR^-^) and ER positive (ER^+^/PR^+^), and intermediate (ER^-^/PR^+ ^or ER^+^/PR^-^) status. This analysis revealed that IL-1β, IL-2, IL-6, IL-8, IL-10, IL-12, IFN-γ, MCP-1, MIP-1β and TNF-α were inversely correlated with true ER status. The most abundant cytokines in true ER negative breast carcinoma were IL-8, MCP-1 and MIP-1β.

### Correlation with other clinical parameters

We then evaluated whether cytokine expression could be linked to clinical parameters such as tumour size, lymph node status, histological grade, or patient age. The cytokine profile was not linked to tumour size or to histological type. We did not observe any correlation between the age of patients at cancer diagnosis and cytokine expression, except for IL-1β, which was inversely correlated with the age of the patient: at age 50 years or younger the median IL-1β level was 3.14 fg/μg (range 0.2 to 194.2 fg/μg); at age 50–65 years the median IL-1β level was 2.13 fg/μg (range 0 to 42.4 fg/μg); and at age above 65 years the median IL-1β level was 1.9 fg/μg (range 0 to 260.6 fg/μg; *P *= 0.033). Lymph node status was not linked to any of the analyzed cytokines.

Because ER-negative tumours are generally of higher grade than ER-positive ones (which was confirmed in our samples; data not shown), we determined whether cytokine levels could be correlated with grade. Most of the cytokines that were preferentially present in ER-negative tumours (IL-1β, IL-6, IL-8, IL-10, IL-12, MCP-1 and MIP-1β) were also more abundant in high-grade tumours (Table 3).

Another factor that is frequently linked to grade and ER status is the HER2 level. We observed a correlation between HER2 levels and IL-8 and MIP-1β, which were expressed to a greater degree in HER2-positive patients; the median (range) IL-8 levels in HER2-negative patients and HER2-positive patients were 50.8 fg/μg (0 to 15,890 fg/μg) and 192.6 fg/μg (23.7 to 8,357 fg/μg), respectively (*P *= 0.014); and for MIP-1β the median (range) levels were 514.9 fg/μg (42.6 to 14,439 fg/μg) and 976.1 fg/μg (44.6 to 4,978 fg/μg), respectively (*P *= 0.021). These data suggest that high cytokine levels are associated with poor prognostic factors such as high grade, ER-negative status, and HER2-positive tumours.

AP-1 transcription factors are known to regulate the expression of many cytokines. To address this issue, we performed gel shift assays measuring global AP-1 binding to specific DNA-binding sites. As reported by others [11], AP-1 was more abundant in ER-negative tumour samples than in ER-positive ones (*P *< 0.001). There was also a higher level of HER2 in AP-1-positive tumours (*P *= 0.025). In contrast, AP-1 status was not correlated with PR expression, grade, size of the tumour and lymph node status. Interestingly, high AP-1 levels correlated with high levels of expression of several cytokines, including IL-1β, IL-2, IL-6, IL-8, IL-10, IL-12, IFN-γ, MCP-1 and TNF-α (Table 4). Among these cytokines, the greatest differences were observed for IL-8 and MCP-1, which respectively exhibited 3.9-fold and 2.7-fold higher expression in AP-1-positive tumours than in AP-1-negative ones (Table 4).

### Tumors expressing high levels of IL-8 exhibit high vascularization

We next focused our attention on the possible association between high expression of IL-8 and vascularization. We analyzed 10 tumours exhibiting high levels of IL-8 and 10 tumours with low IL-8 content. A microscopic analysis showed that tumours with high expression of IL-8 were also more vascularized (median of 125 vessels/mm^2^) than were tumours with low IL-8 content (48 vessels/mm^2^; *P *= 0.0002; Table 5). Overall, these results suggest that IL-8 expression is associated with high neovascularization.

### Leucocyte infiltration is correlated with high levels of cytokines

**Figure 1:**
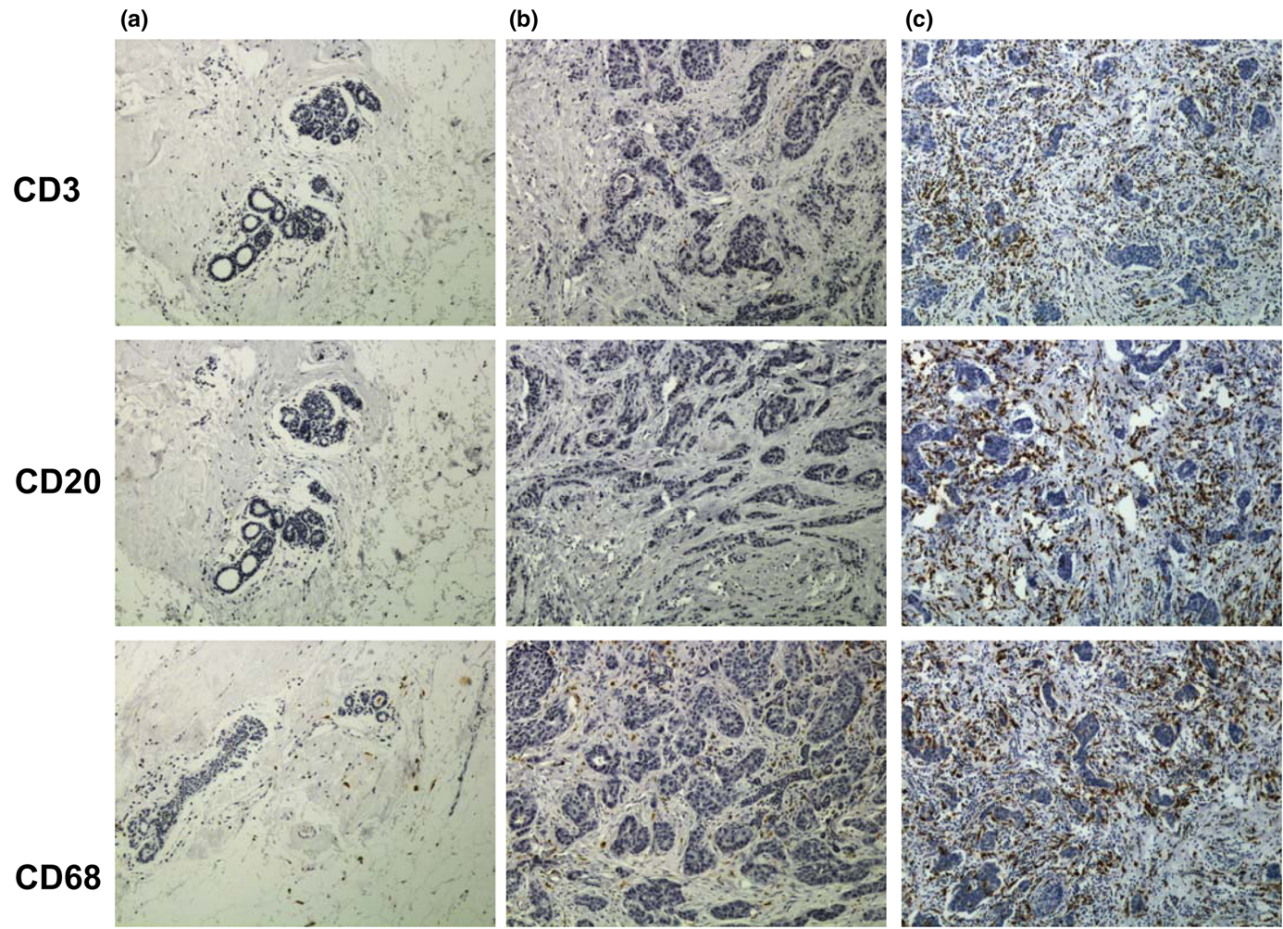
**Breast tumours exhibit various levels of leucocyte infiltration**. Normal breast and breast carcinoma were stained for B lymphocytes, T lymphocytes and macrophages using CD3, CD20 and CD68 immunostaining. Representative pictures of **(a) **normal breast, **(b) **tumour with weak leucocyte infiltration and **(c) **tumour with robust leucocyte infiltration are shown here.

Because IL-8, MCP-1 and MIP-1β, the most highly expressed chemokines in cancer tissues, are potent chemotactic molecules, we investigated whether their expression could be correlated with tumour leucocyte infiltration. We conducted a complete immunohistochemical analysis of our collective of tumour samples using CD3 (B lymphocytes), CD20 (T lymphocytes) and CD68 (macrophages) antibodies. A representative image is shown in Figure 1. Normal breast exhibited no to low staining, whereas breast cancer tissues exhibited a complete range of leucocyte infiltration from low to high levels. Our findings indicate that high levels of IL-8 were correlated with high macrophage infiltration, whereas strong expression of MCP-1 and MIP-1β was correlated with robust presence of B lymphocytes, T lymphocytes and macrophages (Tables 6, 7, 8).

## Discussion

It is well known that the interactions of tumour cells with their microenvironment may affect tumour growth and metastasis formation. Among these, inflammatory cells and cytokines were recently suggested to play a key role in breast carcinoma (for review, see Wilson and Balkwill [4]).

A previous study of breast cancer patients [12] showed that IL-8 RNA was more abundant in the neoplastic than in the normal population, whereas no difference was observed for IL-1α, IL-1β, IL-4, or IL-6. In addition, transcripts for IL-2, IL-4, IL-5, IL-7, TNF-α and IFN-γ were not detected in either group [12,13]. Increased serum levels of the cytokines IL-6, IL-8 and IL-10 have also been observed in patients suffering from breast cancer as compared with healthy women [14,15]. Among the 17 cytokines we analyzed, eight were not detected in normal breast (IL-1β, IL-2, IL-4, IL-5, IL-10, IL-17, G-CSF and GM-CSF) and three were absent from breast carcinoma (IL-5, IL-17 and GM-CSF). With the exception of IL-7, all cytokines that were present in both tissues were significantly overexpressed in breast carcinoma.

To date, the correlation between cytokine expression and clinical parameters remains elusive. We observed that IL-1β, IL-6, IL-8, IL-10, IL-12, MCP-1 and MIP-1β correlated with grade. IL-8 and MIP-1β were also linked to HER2 status, which is in agreement with reports demonstrating that IL-8 is regulated by HER2 in breast cancer cell lines [16]. In addition, IL-1β was inversely correlated to age. IL-1β was previously shown to be strongly expressed in high-grade invasive breast carcinoma as compared with *in situ *ductal carcinoma or benign lesions [17]. In contrast to these results, Green and coworkers [12] did not observe any correlation between the cytokines IL-1α, IL-1β, IL-4, IL-6, IL-8, TNF-α, TNF-β, IL-2, IL-5, IL-7 and IFN-γ and tumour histological grade, patient age or lymph node metastasis in breast cancer patients, but that study analyzed only RNA levels.

Of particular note, in our study we found an inverse correlation between expression of ER and cytokines, which is in agreement the findings of other studies [8,18]. An inverse correlation between IL-8 and ER or PR in breast cancer biopsies has also been demonstrated [19]. Several studies have reported that IL-1α, IL-1β, or IL-6 levels correlated inversely with ER levels [20-22]. This inverse correlation between cytokines and ER status not only may reflect the greater aggressiveness of this subtype of breast tumours but it could also be the result of a direct regulation of cytokine expression by ER. Several reports have demonstrated a direct downregulation of cytokines by ER in different organs. This is not only the case for IL-6 [23,24] and IL-8 [8,18] but also for IL-1β (for review, see Pfeilschifter and coworkers [24]), IL-2 [25], IL-10 [26], IL-12 (for review, see Salem [27]), GM-CSF [24], IFN-γ [28,29], MCP-1 [30,31] and TNF-α [24]. PR is also known to downregulate the expression of a number of cytokines, including IL-1β [32], IL-6 [30,33], IL-8 [34], MCP-1 [32,35] and TNF-α [32], which is in agreement with our findings.

According to our work, Th1 cytokines (IL-2 and IFN-γ) and Th2 cytokines (such as IL-4 and IL-5) are not the major cytokines produced by tumours. However, several cytokines produced by monocytes (IL-1β, IL-6 and MCP-1) or macrophages (such as IL-1β, IL-6, IL-8, MIP-1β and TNF-α) are highly expressed by the tumour. We observed that tumours displaying high levels of IL-8 exhibited significantly greater vascularization. A role for IL-8 in breast cancer angiogenesis has also been suggested by *in vitro *studies [18], but to our knowledge ours is the first study to report an association between IL-8 levels and angiogenesis in breast cancer samples.

We found that tumours expressing high levels of MCP-1 and MIP-1β had greater B lymphocyte, T lymphocyte and macrophage content than did tumours expressing low levels of these chemokines. Similarly, robust expression of IL-8 was correlated with marked macrophage infiltration. Our results are thus in agreement with previous studies that showed a correlation between the number of tumour associated macrophages and IL-6, IL-8 or MCP-1 levels in breast and cervical cancers [36-38]. On the other hand, the correlation between MIP-1β and macrophage infiltration has not been reported to date, or has the association of high levels of MCP-1 and MIP-1β with the presence of B and T lymphocytes in breast carcinoma. The precise role played by leucocytes in tumours remains to be elucidated in larger studies, which may determine whether they contribute to tumour growth.

## Conclusion

The pattern of expression of multiple cytokines in breast cancer tissues that we report is the first step in elucidating the involvement of cytokines in breast cancer. Our data suggest that cytokines could be involved in the aggressiveness of ER-negative breast tumours. In addition, robust expression of IL-8, MCP-1 and MIP-1β, which are the three major chemokines present in breast tumours, is correlated with marked leucocyte infiltration. We therefore believe that our work provides justification for further studies to determine more precisely the role played by these cytokines in breast cancer.

## Abbreviations

AP = activator protein; ER = oestrogen receptor; G-CSF = granulocyte colony-stimulating factor; GM-CSF = granulocyte-macrophage colony-stimulating factor; IL = interleukin; IFN = interferon; MCP = monocyte chemoattractant protein; MIP = macrophage inflammatory protein; PR = progesterone receptor; Th = T-helper (cell); TNF = tumour necrosis factor.

## Competing interests

The authors declare that they have no competing interests.

## Authors' contributions

CC carried out the bioplex experiments and helped to draft the manuscript. FB interpreted the immunhistochemistry slides and revised the manuscript. SGB performed all stastical analysis and revised the manuscript. SB, SR and FB performed the immunohistochemistry experiments and FB also revised the manuscript. DL was involved in the bioplex experiments. GL conceived the study, supervised the work, drafted the manuscript, processed the biopsy samples, and performed the bioplex and gel shift experiments.

## Supplementary Material

Additional file 1 A Word document containing a table that summarizes the clinical characteristics of the patients. Click here for file

Additional file 2 A word document containing a summary of the manufacturer's instructions on the cytokine multiplexed bioplex assay. Click here for file

Additional file 3 A pdf file including a figure the compares IL-8 concentration measured by bioplex assay and by conventional enzyme-linked immunosorbent assay (*n *= 93). Click here for file

Additional file 4 A pdf file including a table that summarizes the distribution of cytokines by hormone receptor status. Click here for file

## Figures and Tables

**Table 1:** Cytokines are overexpressed in breast cancer

Biological markers	Median protein (range; fg/μg)		*P*
		
	Cancer (*n *= 105)	Healthy breast (*n *= 13)	
IL-1β	2.7 (0–260)	0	< 0.0001
IL-2	1.2 (0–42.6)	0	< 0.0001
IL-4	1.7 (0–99.6)	0	< 0.0001
IL-5	0	0	
IL-6	17.2 (4.4–26,487)	1.2 (0–52.5)	< 0.0001
IL-7	0.7 (0–8.5)	1.1 (0.7–16.9)	0.047
IL-8	68.1 (0–15,890)	1 (0–78.5)	< 0.0001
IL-10	0.3 (0–5.6)	0	< 0.0001
IL-12 (p70)	2.3 (0–19.3)	1.4 (0.5–20.4)	0.005
IL-13	4.2 (1.6–394)	1.9 (1.2–21.6)	< 0.0001
IL-17	0	0	
G-CSF	18.7 (5.3–110)	0 (0–49.2)	< 0.0001
GM-CSF	0	0	
IFN-γ	27.6 (15.5–1,279)	16.6 (13.6–187)	< 0.0001
MCP-1	121 (0–37117)	1.9 (0–45.9)	< 0.0001
MIP-1β	559 (42.6–14,439)	8.3 (5.0–142)	< 0.0001
TNF-α	7.2 (5.8–121)	1.6 (1.3–23.2)	< 0.0001

G-CSF, granulocyte colony-stimulating factor; GM-CSF, granulocyte-macrophage colony-stimulating factor; IL, interleukin; IFN, interferon; MCP, monocyte chemoattractant protein; MIP, macrophage inflammatory protein; TNF, tumour necrosis factor.

**Table 2:** Cytokines are present at high levels in ER-negative and PR-negative breast tumors

Biological marker	ER negative (*n *= 45)		ER positive (*n *= 60)		*P*	PR negative (*n *= 30)		PR positive (*n *= 75)		*P*
IL-1β	3.7	(0–260)	2.2	(0–42.4)	0.031	5.3	(0–194)	2.3	(0–260)	0.008
IL-2	1.7	(0–42.6)	0.9	(0–11.0)	0.003	1.7	(0–6.6)	1.2	(0–42.6)	0.119
IL-4	3.8	(0–20.0)	1.1	(0–99.6)	NS	4.6	(0–18.7)	1.1	(0–99.6)	0.040
IL-6	25.4	(7.8–26,487)	13.2	(4.4–619)	< 0.001	53.6	(9.9–26,487)	13.7	(4.4–4,312)	< 0.001
IL-8	158	(5.0–15,890)	40.5	(0–10,298)	< 0.001	505	(7.3–15,890)	48.7	(0–11,281)	< 0.001
IL-10	0.6	(0–5.6)	0.2	(0–2.86)	0.003	0.8	(0–5.0)	0.2	(0–5.6)	< 0.001
IL-12 (p70)	3.0	(0 -19.3)	2.0	(0–17.2)	0.029	3.1	(0–7.3)	2.0	(0–19.3)	0.024
IL-13	4.3	(3.4–52.6)	4.1	(1.6–394)	0.049	4.3	(3.6–35.5)	4.1	(1.6–394)	NS
G-CSF	17.3	(7.1–110)	20.1	(5.3–87.3)	NS	24.1	(7.1–110)	17.4	(5.3–103)	0.056
IFN-γ	32.6	(19.4–324)	25.6	(15.5–1,279)	< 0.001	33.8	(23.7–324)	25.6	(15.5–1,279)	0.002
MCP-1	210	(14.7–37,117)	80.7	(0–1,543)	< 0.001	404	(17.7–37,717)	96.3	(0–1,543)	< 0.001
MIP-1β	748	(42.6–14,439)	488	(87.2–10,244)	0.005	1121	(44.6–13,287)	493	(42.6–14,439)	0.001
TNF-α	8.5	(6.1–80.0)	6.9	(5.8–120)	< 0.001	8.6	(6.2–37.2)	7.1	(5.8–121)	0.002

Values are expressed as median (range; fg/μg). G-CSF, granulocyte colony-stimulating factor; IL, interleukin; IFN, interferon; MCP, monocyte chemoattractant protein; MIP, macrophage inflammatory protein; NS, not significant; TNF, tumour necrosis factor.

**Table 3:** Most cytokines are abundant in high-grade tumors

Biological markers	Grade I (*n *= 17)		Grade II (*n *= 56)		Grade III (*n *= 32)		*P*
IL-1β	0.8	(0–260)	2.6	(0–102)	4.7	(0–194)	0.025
IL-2	0.9	(0–4.6)	1.2	(0–42.6)	1.4	(0–6.6)	NS
IL-4	3.4	(0–20.1)	0.6	(0–30.2)	3.9	(0–99.6)	0.037
IL-6	11.7	(7.8–4,312)	14.1	(4.4–190)	30.6	(8.8–26,487)	< 0.001
IL-8	43.9	(9.1–11,281)	35.4	(0–8,358)	186	(0.3–15,890)	< 0.001
IL-10	0.2	(0–1.6)	0.2	(0–5.6)	0.7	(0–4.6)	0.030
IL-12 (p70)	1.4	(0–5.3)	2.1	(0–19.3)	2.9	(0.7 -17.2)	0.045
IL-13	4.1	(3.4–21.4)	4.2	(3.0–394)	4.2	(1.6–35.5)	NS
G-CSF	13.9	(7.1–87.3)	18.9	(5.3–110)	22.2	(9.9–79.2)	NS
IFN-γ	26.8	(19.8–270)	25.6	(17.1–1,279)	28.7	(15.5–324)	NS
MCP-1	96.3	(22.8–1,208)	91.3	(0–22,451)	201	(24.4–37,117)	0.006
MIP-1β	451	(44.6–2,674)	496	(42.6–14,439)	776	(114–13,287)	0.011
TNF-α	7.15	(5.9–28.0)	7.1	(5.8–120)	7.8	(6.2–37.2)	NS

Values are expressed as median (range; fg/μg). G-CSF, granulocyte colony-stimulating factor; IL, interleukin; IFN, interferon; MCP, monocyte chemoattractant protein; MIP, macrophage inflammatory protein; NS, not significant; TNF, tumour necrosis factor.

**Table 4:** Cytokine levels are correlated to AP-1 expression

Biological markers	AP-1 negative (*n *= 34)		AP-1 positive (*n *= 69)		*P*
IL-1β	1.4	(0–11.5)	3.2	(0–260)	0.006
IL-2	0.4	(0–3.2)	1.4	(0–11)	0.001
IL-4	1.1	(0–99.6)	2.7	(0–30.2)	NS
IL-6	12.6	(4.4–70.7)	19.7	(7.8–26,487)	0.001
IL-8	29.6	(0 -778)	115	(1.5–15,890)	0.003
IL-10	0.2	(0–2.6)	0.3	(0– 5.6)	0.004
IL-12 (p70)	1.8	(0 -17.2)	2.7	(0–7.3)	0.044
IL-13	4.2	(3.0–7.2)	4.2	(3.4–394)	NS
G-CSF	17.2	(5.3–87.3)	18.5	(7.1–110)	NS
IFN-γ	25.4	(15.5–55.5)	28.7	(17.1–1,279)	0.002
MCP-1	54.6	(0–1,208)	148	(14.7–37,117)	0.001
MIP-1β	503	(87.2–8,778)	563	(42.6–14,439)	NS
TNF-α	6.9	(6.1–10.9)	7.8	(5.8–121)	0.002

Values are expressed as median (range; fg/μg). AP, activator protein; G-CSF, granulocyte colony-stimulating factor; IL, interleukin; IFN, interferon; MCP, monocyte chemoattractant protein; MIP, macrophage inflammatory protein; NS, not significant; TNF, tumour necrosis factor.

**Table 5:** IL-8 expression is correlated to angiogenesis

IL-8	Vessels/mm^2^
-	48
-	72
-	53
-	44
-	30
-	ND
-	57
-	46
-	63
-	20
+	244
+	128
+	78
+	279
+	116
+	209
+	124
+	94
+	81
+	359

Vessel density in a series of tumors with high (+; *n *= 10) and low (-; *n *= 10) expression of IL-8. Haematoxylin and eosin saffron stain was used. ND, not detectable.

**Table 6:** IL-8 expression and leucocyte infiltration

Antibody	Staining	*n*	IL-8 median^a^	IL-8 minimum^a^	IL-8 maximum^a^	*P*^b^
CD3	0	54	47.6	0	11,281	0.17
	1	36	67.7	0.3	15,890	
	2	11	289.7	27.6	10,298	
	3	4	145.0	14.9	4,408	
CD20	0	74	59.2	0	15,890	0.42
	1	27	219.9	1.5	10,298	
	2	3	165.2	14.9	4,408	
	3	1	124.8	124.8	124.8	
CD68	0	5	23.6	0.6	146.7	0.0011
	1	59	48.6	0	15,890	
	2	32	108.6	5.6	11,281	
	3	9	289.9	14.9	10,298	

Values are expressed as fg/μg. ^b^By analysis of variance. IL, interleukin.

**Table 7:** MCP-1 expression and leucocyte infiltration

Antibody	Staining	*n*	MCP-1 median^a^	MCP-1 minimum^a^	MCP-1 maximum^a^	*P*^b^
CD3	0	54	110.5	0	1,543	0.0001
	1	36	107.9	23.3	8,479	
	2	11	278.3	45.5	22,451	
	3	4	3307	196.5	37,117	
CD20	0	74	110.3	0	8,478	0.0002
	1	27	192.9	23.35	22,451	
	2	3	3185	196.5	37,117	
	3	1	3439	3430	3,430	
CD68	0	5	40.9	7.0	167.4	0.0016
	1	59	102.6	8.0	8,479	
	2	32	137.9	0	37,117	
	3	9	478.3	45.5	3,430	

Values are expressed as fg/μg. ^b^By analysis of variance. MCP, monocyte chemoattractant protein.

**Table 8:** MIP-1β expression and leucocyte infiltration

Antibody	Staining	*n*	MIP-1β median^a^	MIP-1β minimum^a^	MIP-1β maximum^a^	*P*^b^
CD3	0	54	472.5	42.6	12835	< 0.0001
	1	36	560.7	44.6	10685	
	2	11	2702	274.5	14439	
	3	4	6757	1226	13287	
CD20	0	74	495.9	42.6	14439	< 0.0001
	1	27	745.3	44.6	10685	
	2	3	4394	1226	9121	
	3	1	13287	13287	13287	
CD68	0	5	342.4	223.4	498.7	< 0.0001
	1	59	458	42.6	12835	
	2	32	739.8	134.3	14439	
	3	9	3095	589.3	13287	

Values are expressed as fg/μg. ^b^By analysis of variance. MIP, macrophage inflammatory protein.
