# Supplementary material for: Oestrogen receptor negative breast cancers exhibit high cytokine content
Source: Breast Cancer Res. 2007 Jan 29;9(1):R15. doi: 10.1186/bcr1648 (PMC1851386; doi:10.1186/bcr1648)
Supplement: Additional file 1 — A Word document containing a table that summarizes the clinical characteristics of the patients. [file bcr1648-S1.doc]

**Table S1 Clinical characteristics of patients**

| Patient characteristics | No |
| --- | --- |
| Total Population | 105 |
| Median Age (range) | 59 (33-89) |
| Estrogen receptor (ER) |  |
| Positive | 60 |
| Negative | 45 |
| Progesterone receptor (PR) |  |
| Positive | 75 |
| Negative | 30 |
| Primary tumor |  |
| T1 (≤ 2cm) | 31 |
| T2 (> 2-5 cm) | 63 |
| T3 (> 5 cm) | 5 |
| T4 | 4 |
| Unknown | 2 |
| Regional Lymph nodes |  |
| Negative | 48 |
| Positive | 50 |
| Unknown | 7 |
| Histologic grade |  |
| I | 17 |
| II | 56 |
| III | 32 |
| HER2 |  |
| Negative | 90 |
| Positive | 14 |
| Histologic Type |  |
| Infitrating ductal | 79 |
| Infiltrating Lobular | 19 |
| Mixt | 3 |
| Others | 4 |
|  |  |

**Table S2 Cytokines are highly expressed in true ER-negative breast tumors**

| Median Biological Markers fg/µg protein (range | | | | | |  |  | |  |  |
| --- | --- | --- | --- | --- | --- | --- | --- | --- | --- | --- |
|  | ER- / PR- (n=21) | |  | ER- /PR+ or ER+ / PR- (n=30) | |  | ER+ /PR+ (n=51) | |  | p |
|  |  |  |  |  |  |  |  |  |  |  |
| IL-1β | 10.8 | (0 - 194) |  | 1.6 | (0 - 260) |  | 2.4 | (0- 11.5) |  | 0.001 |
| IL-2 | 2.03 | (0 - 6.6) |  | 1.2 | (0 - 42.6) |  | 0.9 | (0 - 11.0) |  | 0.022 |
| IL-4 | 4.9 | (0 - 18.7) |  | 1.7 | (0 - 20.1) |  | 1.0 | (0 - 99.6) |  | 0.081 |
| IL-6 | 63.8 | (10.8 - 26,487) |  | 17.2 | (7.8 - 4,312) |  | 13.1 | (4.4 - 95.6) |  | <0.001 |
| IL-8 | 639.7 | (7.3 - 15,890) |  | 69.7 | (5.0 - 11,281) |  | 40.5 | (0 - 491) |  | <0.001 |
| IL-10 | 0.99 | (0 - 5.0) |  | 0.26 | (0 - 5.6) |  | 0.20 | (0 - 2.9) |  | <0.001 |
| IL-12 (p70) | 3.2 | (0 - 7.3) |  | 2.3 | (0 - 19.3) |  | 2.0 | (0 - 17.2) |  | 0.014 |
| IL-13 | 4.3 | (3.6 - 35.5) |  | 4.2 | (3.4 - 52.6) |  | 4.1 | (1.6 - 394) |  | NS |
| G-CSF | 24.3 | (7.1 - 110) |  | 16.2 | (8.7 - 103) |  | 19.7 | (5.3 - 87.3) |  | NS |
| IFNγ | 33.8 | (23.7 - 324) |  | 27.6 | (19.4 - 278) |  | 25.4 | (15.5 - 1,279) |  | <0.001 |
| MCP-1 | 817.4 | (30.5 - 37,117) |  | 121.5 | (14.7 - 799) |  | 80.7 | (0 - 1,543) |  | <0.001 |
| MIP-1β | 2,702 | (44 - 13,287) |  | 493 | (42 - 14,439) |  | 488 | (87 - 10,244) |  | <0.001 |
| TNFα | 10.0 | (6.4 - 37.2) |  | 7.1 | (6.1 - 80.0) |  | 7.0 | ( 5.8 - 120.8) |  | <0.001 |
|  |  |  |  |  |  |  |  |  |  |  |
