# Supplementary material for: Oestrogen receptor negative breast cancers exhibit high cytokine content
Source: Breast Cancer Res. 2007 Jan 29;9(1):R15. doi: 10.1186/bcr1648 (PMC1851386; doi:10.1186/bcr1648)
Supplement: Additional file 2 — A word document containing a summary of the manufacturer's instructions on the cytokine multiplexed bioplex assay. [file bcr1648-S2.doc]

**Cytokine multiplexed Bioplex assay (Manufacturer Instructions)**

Each well of the 96-well filter plate was prewet with 100 µl of Bioplex assay Buffer A (provided by the manufacturer). The buffer was then removed by vacuum filtration. For each well, the multiplex bead stock was prepared by mixing 2 µl of anti-cytokine conjugated beads with 48 µl of Bioplex assay Buffer A. 50 µl of multiplex bead stock was added to each well and then vacuum filtration was applied. 100 µl of Bioplex wash Buffer A (provided by the manufacturer) was added to each well and then removed by vacuum filtration. 50 µl of diluted standards or samples were added to each well. For each cytokine, 8 standards ranging from 2 to 32,000 pg/ml were used. The filter plate was then shaked at room temperature at 1100 rpm for 30 sec, then at 300 rpm for 30 min. The buffer was then removed by vacuum filtration. Wells were then washed 3 times with 100 µl of Bioplex wash Buffer A. 25 µl of cytokine detection antibody solution diluted in detection antibody diluent A was added to each well. The filter plate was then shaked at room temperature at 1100 rpm for 30 sec, then at 300 rpm for 30 min. The buffer was removed by vacuum filtration. Wells were washed 3 times with 100 µl of Bioplex wash Buffer A. 50 µl of streptavidin-PE solution were added to each well. The filter plate was shaked at room temperature at 1100 rpm for 30 sec, then at 300 rpm for 10 min. Wells were washed 3 times with 100 µl of Bioplex wash Buffer A. Beads were then resuspended with 125 µl of Bioplex assay Buffer A, shaked at 1100 rpm for 30 sec. The filter plate was read on a Bioplex reader.
