# Supplementary figures and images for: Oestrogen receptor negative breast cancers exhibit high cytokine content
Source: Breast Cancer Res. 2007 Jan 29;9(1):R15. doi: 10.1186/bcr1648 (PMC1851386; doi:10.1186/bcr1648)

Comparison between Elisa And Bioplex results for IL-8

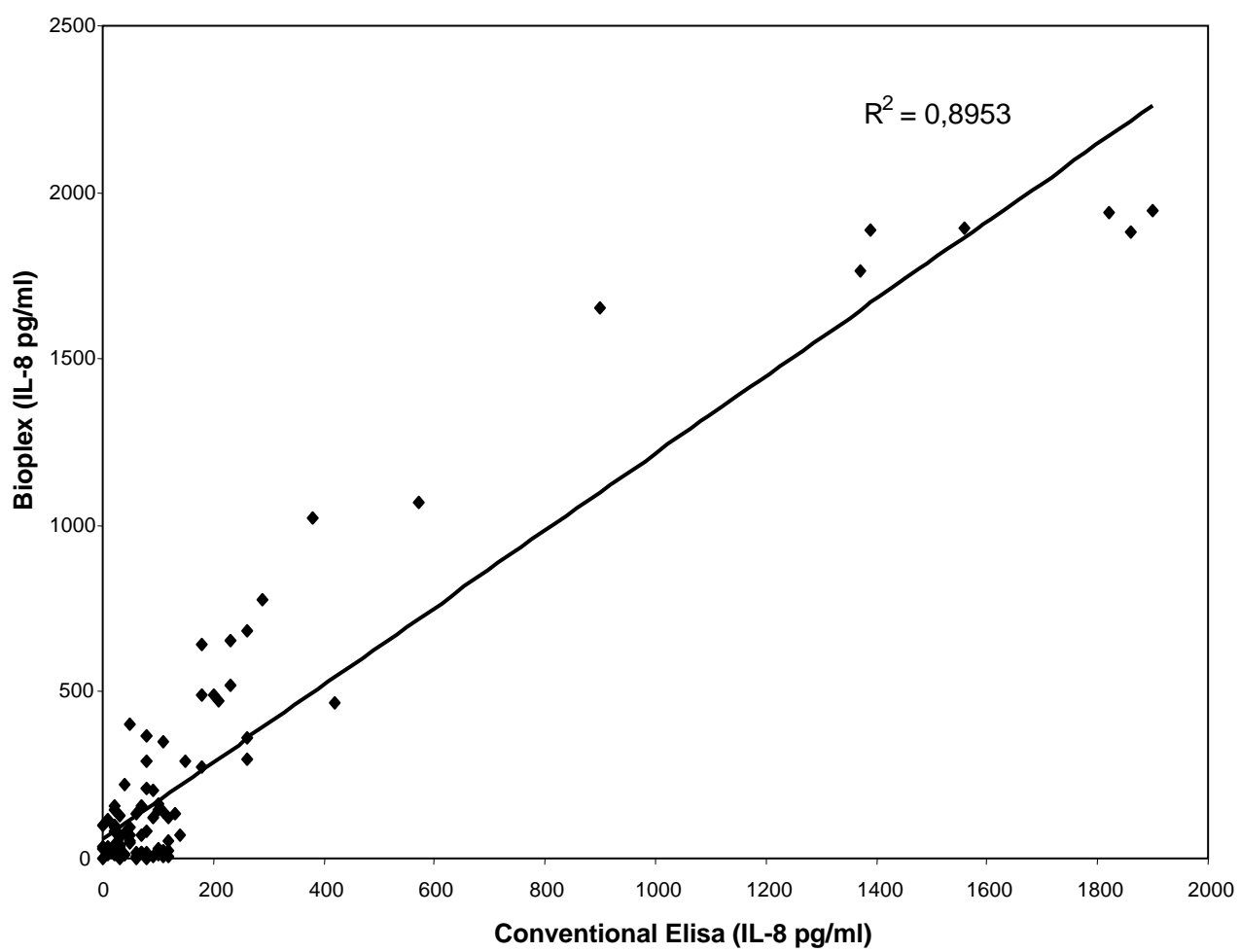

Supplement: Additional file 3 — A pdf file including a figure the compares IL-8 concentration measured by bioplex assay and by conventional enzyme-linked immunosorbent assay (n = 93). [file bcr1648-S3.pdf]
