# Supplementary material for: Oestrogen receptor negative breast cancers exhibit high cytokine content
Source: Breast Cancer Res. 2007 Jan 29;9(1):R15. doi: 10.1186/bcr1648 (PMC1851386; doi:10.1186/bcr1648)
Supplement: Additional file 4 — A pdf file including a table that summarizes the distribution of cytokines by hormone receptor status. [file bcr1648-S4.pdf]

Patient	AGE	GRADE	SIZE	N	ER	PR	HER-2	hIL-1b	hIL-2	hIL-4	hIL-5	hIL-6	hIL-7	hIL-8	hIL-10	hIL-12	hIL-13	hIL-17	hG-CSF	hGM-CSF	hIFNg	hMCP-1	hMIP1-b	hTNFa	AP-1	CD3	CD20	CD68
1	49	III	T2	N0	neg	neg	0	58.08	5.20	8.33	0.00	220.39	6.68	8867.98	2.62	7.20	4.93	7.31	24.04	43.07	61.35	4027.72	10685.01	18.13	pos	1	1	2
2	37	II	T3	N1	neg	neg	3+	101.64	5.49	9.27	0.00	101.85	0.54	8357.71	4.99	6.05	4.46	4.15	47.43	0.00	64.07	22451.66	3257.62	14.89	pos	2	1	2
3	67	I	T2	N0	neg	neg	0	7.14	0.87	6.23	0.00	13.38	0.23	363.04	0.40	2.34	3.93	0.42	14.32	0.00	26.78	277.33	2673.97	7.51	neg	0	0	1
4	38	III	T1	N0	neg	neg	0	17.17	2.89	4.87	0.00	3304.14	1.48	6628.85	1.31	4.13	4.53	2.72	31.72	102.44	43.10	830.17	1285.22	11.83	pos	0	0	1
5	33	I	T1	N1	neg	pos	0	2.32	1.16	7.70	0.00	20.98	1.53	71.07	0.17	3.37	4.60	0.00	13.64	0.00	32.61	288.55	743.72	8.05	pos	1	1	1
6	59	III	T1	N0	neg	neg	1+	6.13	2.32	0.00	0.00	29.50	1.57	289.74	0.96	3.11	3.86	0.35	27.20	0.00	26.40	192.93	4978.47	7.78	pos	2	1	2
7	45	III	T2	N0	neg	neg	0	14.40	1.74	2.36	0.00	129.27	0.67	493.25	2.01	3.75	4.33	0.35	27.99	0.00	33.78	4996.57	4011.84	15.07	pos	1	1	2
8	50	II	T2	N1	neg	pos	0	1.22	2.60	11.68	0.00	18.79	0.09	14.90	5.60	4.13	4.60	0.20	17.71	0.00	39.21	196.50	1226.75	8.77	pos	3	2	3
9	48	III	T2	N0	neg	neg	0	14.48	5.20	6.55	0.00	26487.47	1.35	15890.60	0.64	3.24	4.40	4.01	79.18	85.31	91.26	8479.38	114.38	20.74	pos	1	0	1
10	67	III	T2	N1	neg	neg	0	0.00	0.00	3.93	0.00	48.97	0.76	31.52	0.11	1.58	3.93	0.00	16.58	0.00	25.23	46.30	460.90	6.43	pos	1	0	2
11	52	III	T3	N1	neg	pos	3+	2.48	1.16	3.72	0.00	19.71	1.66	366.33	0.38	2.47	3.53	0.49	17.03	0.00	28.34	175.84	731.32	6.70	pos	0	0	2
12	65	III	T2	N1	neg	neg	3+	0.00	0.29	0.00	0.00	69.94	1.08	219.89	0.11	1.19	3.79	0.00	11.83	0.00	23.29	88.12	335.19	6.25	pos	1	1	2
13	81	I	T1	N0	neg	pos	0	0.92	4.05	1.73	0.00	34.11	1.30	115.18	0.61	3.24	21.41	1.21	14.77	0.00	270.31	256.68	342.09	28.03	pos	0	0	1
14	37	III	T2	N1	neg	neg	0	11.28	6.65	5.92	0.00	49.20	2.33	124.81	4.43	5.15	14.65	5.52	14.20	0.00	72.23	3429.85	13287.59	18.58	pos	3	3	3
15	49	II	T2	N1	neg	pos	0	1.59	7.40	0.00	0.00	15.46	2.57	50.38	0.87	6.61	7.23	0.52	15.95	0.00	46.19	72.80	14439.64	11.85	pos	2	0	2
16	89	II	T2	Nx	neg	neg	0	9.22	1.74	9.06	0.00	13.49	1.17	15.99	0.35	3.49	4.06	0.00	65.39	0.00	24.84	128.41	580.64	7.15	pos	0	0	2
17	62	II	T1	N1	neg	pos	0	5.06	4.05	0.00	0.00	25.59	2.24	160.37	0.11	3.37	5.07	0.00	17.26	0.00	113.01	532.08	755.59	16.33	pos	0	0	1
18	59	I	T2	N1	neg	pos	0	0.51	0.87	7.81	0.00	7.85	0.09	158.51	0.17	0.68	3.39	0.00	9.57	0.00	22.12	65.43	522.50	6.97	pos	0	0	1
19	74	II	T2	N1	neg	pos	0	3.03	0.29	0.00	0.00	11.76	0.27	17.53	0.14	2.34	3.99	0.00	24.60	0.00	25.23	54.21	493.05	6.61	pos	0	0	1
20	41	II	T2	N0	neg	pos	0	4.13	4.92	1.10	0.00	12.57	1.79	146.76	0.40	3.49	11.57	0.28	31.61	0.00	62.13	167.43	367.71	9.94	pos	0	0	0
21	40	III	T2	N1	neg	neg	3+	10.90	1.45	10.01	0.00	18.22	0.94	1432.79	2.89	2.73	4.20	1.35	24.26	0.00	34.94	397.44	2520.89	7.78	pos	1	0	2
22	48	II	T2	N1	neg	pos	0	0.81	1.74	0.47	0.00	23.40	0.09	38.65	0.17	1.45	15.99	17.51	12.40	0.00	56.30	112.09	265.10	8.68	pos	1	0	2
23	47	II	T2	N1	neg	pos	3+	4.37	1.74	0.00	0.00	17.64	0.90	98.87	0.58	1.70	4.20	0.00	14.54	0.00	31.45	160.29	902.92	7.78	pos	1	0	1
24	71	II	T2	N1	neg	neg	0	0.00	0.01	1.41	0.00	14.87	0.45	7.26	0.29	0.94	3.59	0.00	14.77	0.00	23.68	30.49	434.54	6.79	pos	0	0	1
25	49	III	T2	N1	neg	neg	1+	10.81	1.74	0.00	0.00	44.25	0.00	165.25	3.94	4.00	4.26	0.00	27.20	0.00	33.78	3184.93	4394.13	10.03	pos	3	2	2
26	88	I	T2	N0	neg	pos	0	260.65	4.63	20.07	0.00	4312.47	0.81	11281.86	1.63	5.28	4.87	5.52	56.81	304.50	83.10	799.06	903.98	21.19	pos	0	0	2
27	79	II	T2	N1	neg	neg	0	3.66	1.16	4.35	0.00	150.58	1.84	1019.37	0.90	2.98	3.86	0.99	15.11	0.00	36.49	817.42	3116.26	10.75	pos	1	1	1
28	52	III	T1	N0	neg	neg	0	3.63	2.89	5.08	0.00	113.49	0.23	684.18	0.78	2.85	3.73	0.00	19.97	0.00	29.11	210.01	748.29	8.14	pos	1	1	2
29	47	III	T2	Nx	neg	pos	3+	5.77	0.87	1.41	0.00	14.41	0.23	69.72	0.87	1.70	4.20	0.00	16.13	0.00	27.56	121.53	541.15	7.69	pos	1	0	2
30	70	III	T2	N0	neg	neg	0	4.57	3.47	3.82	0.00	668.63	3.09	7786.86	0.99	5.92	35.48	3.51	25.62	0.00	324.69	2691.51	588.27	36.31	pos	1	0	1
31	56	I	T1	N0	neg	neg	3+	0.00	0.00	0.00	0.00	10.84	0.00	23.69	0.00	0.00	4.13	0.00	7.09	0.00	28.73	43.75	44.61	7.15	pos	1	1	1
32	79	II		N0	neg	neg	3+	23.50	1.74	0.00	0.00	58.07	1.12	289.93	0.99	3.88	7.01	2.22	25.62	0.00	33.78	723.84	3103.89	9.67	pos	1	1	3
33	52	II	T1	N0	neg	pos	0	2.49	42.62	0.08	0.00	93.51	8.04	117.60	4.57	19.28	52.62	46.75	103.13	489.45	277.85	294.12	12835.00	80.00	ND	0	0	1
34	35	II	T1	N1	neg	neg	3+	1.52	0.29	4.77	0.00	21.56	0.81	778.36	0.20	2.09	4.87	0.00	13.30	0.00	32.22	326.29	957.31	7.96	neg	2	0	2
35	76	II	T4	N1	neg	pos	0	0.00	0.00	0.00	0.00	17.18	3.41	5.01	0.00	0.00	3.46	0.00	8.67	0.00	19.40	14.68	42.61	6.07	pos	0	0	1
36	34	III	T1	N0	neg	neg	0	194.20	6.36	18.71	0.00	63.83	3.94	4408.29	4.55	7.33	5.00	5.52	40.87	24.81	77.66	37117.90	9121.34	37.21	pos	3	2	2
37	47	I	T1	N0	neg	pos	0	48.90	1.16	0.00	0.00	11.53	0.23	78.52	0.03	1.45	3.39	0.00	13.87	0.00	29.11	75.37	379.87	8.50	pos	0	0	1
38	43	II	T1	N0	neg	pos	0	2.70	0.01	5.29	0.00	8.31	0.41	5.97	0.08	2.09	3.73	0.00	13.98	0.00	24.07	30.75	478.85	6.43	pos	0	0	1
39	76	II	T2		neg	neg	0	0.35	0.87	4.87	0.00	8.88	0.36	21.76	0.11	1.83	4.40	0.00	23.58	0.00	25.62	20.80	273.50	6.70	pos	0	0	1
40	53	I	T1	N0	neg	pos	0	0.35	0.01	4.87	0.00	9.81	0.36	18.62	0.26	1.32	4.33	0.00	57.48	0.00	25.23	22.84	326.62	7.15	pos	0	0	1
41	81	III	T1	N0	neg	neg	0	0.75	1.35	0.00	0.00	208.13	8.53	3934.77	0.08	1.45	5.25	0.00	15.17	0.00	38.46	504.70	874.13	10.65	pos	0	0	2
42	55	III	T2	N1	neg	pos	0	9.42	2.03	6.97	0.00	147.24	1.70	472.38	1.16	3.11	4.13	2.79	18.72	0.00	30.67	478.27	4137.89	8.59	pos	0	1	3
43	59	II	T1	N0	neg	pos	0	1.09	3.18	1.73	0.00	13.49	1.88	33.06	0.08	2.47	4.33	0.00	13.64	0.00	24.45	127.90	414.67	6.70	pos	0	0	2
44	33	II	T2	N0	neg	neg	0	21.06	3.47	9.17	0.00	190.21	1.26	639.69	4.64	4.64	5.33	0.42	110.14	0.00	50.48	1111.95	2702.39	16.42	pos	2	1	3
45	59	II	T2	N0	neg	neg	ND	0.68	0.29	0.00	0.00	25.36	0.09	132.32	0.35	1.45	3.93	0.00	13.53	0.00	22.90	112.60	103.32	6.88	pos	0	0	1
46	75	II	T2	N0	pos	pos	0	7.22	2.03	2.67	0.00	9.34	0.58	10.59	0.26	1.96	3.86	0.00	17.37	0.00	27.56	103.68	523.56	7.06	pos	1	0	1
47	41	III	T2	N1	pos	pos	2+	0.57	2.03	1.20	0.00	15.68	0.94	139.89	0.14	2.98	3.79	0.00	14.66	0.00	23.68	108.52	2197.76	6.97	pos	0	0	1
48	78	II	T2	N0	pos	neg	0	4.54	0.00	0.00	0.00	29.04	0.32	517.45	0.26	3.88	3.59	0.00	16.24	0.00	27.95	316.09	600.72	7.96	pos	0	0	1
49	54	I	T1	N0	pos	pos	0	1.14	0.29	8.85	0.00	10.27	0.27	43.85	0.05	1.96	3.79	0.00	31.95	0.00	29.89	50.89	365.22	6.88	pos	2	1	2
50	46	II	T2	N1	pos	pos	0	3.20	1.45	6.55	0.00	21.44	0.85	37.69	0.38	2.98	4.46	0.00	10.81	0.00	34.55	152.89	2603.39	8.95	pos	0	0	1
51	66	II	T2	N1	pos	pos	0	5.53	1.74	15.56	0.00	15.34	0.23	5.65	2.86	3.49	3.99	0.00	24.83	0.00	31.06	80.22	2303.18	8.41	pos	1	1	2
52	82	I	T2	N1	pos	pos	0	10.21	1.16	4.14	0.00	18.10	0.18	13.29	0.26	4.39	4.20	0.00	87.32	0.00	31.06	48.34	536.29	8.14	neg	0	0	1
53	60	II	T1	N1	pos	pos	0	5.55	2.03	3.09	0.00	18.91	0.63	66.64	0.17	4.13	4.80	0.00	23.13	0.00	84.66	101.38	562.53	11.20	pos	1	0	2
54	56	II	T2	N0	pos	neg	0	1.42	1.16	2.88	0.00	10.96	0.27	26.07	0.14	1.32	3.73	0.00	14.77	0.00	25.62	40.95	342.37	6.79	pos	0	0	0
55	59	III	T2	N0	pos	neg	0	42.43	2.32	7.39	0.00	619.90	0.63	10298.60	1.84	4.52	4.33	2.29	25.28	0.00	38.44	410.95	3095.18	22.54	pos	2	1	3
56	38	II	T2	N1	pos	pos	0	4.43	0.87	0.00	0.00	8.42	0.41	100.28	0.20	1.96	3.53	0.78	23.13	0.00	24.84	158.50	457.96	7.78	pos	0	0	1
57	71	II	T1	N0	pos	pos	0	3.25	2.03	5.19	0.00	8.19	0.23	5.59	0.23	1.83	4.20	0.00	20.19	0.00	24.07	47.07	558.98	7.33	neg	1	1	1
58	78	II	T4	N0	pos	pos	0	2.18	0.87	0.00	0.00	13.15	1.17	491.58	0.20	2.34	3.73	0.00	20.76	0.00	23.29	308.95	562.81	7.24	pos	0	0	1
59	76	II	T2	N0	pos	pos	0	4.79	2.03	0.00	0.00	29.16	1.57	51.30	1.16	4.13	4.26	0.00	22.68	0.00	30.28	400.75	1019.00	7.42	neg	1	0	2
60	51	III	T1	N0	neg	pos	0	4.76	2.60	5.61	0.00	31.35	1.17	465.77	0.29	2.85	4.66	0.00	22.57	0.00	25.62	242.91	648.41	7.87	pos	0	0	1
61	43	II	T2	N1	pos	pos	0	5.03	2.32	6.23	0.00	17.87	1.21	349.51	2.59	5.41	4.26	1.07	13.30	0.00	31.83	664.68	8778.51	8.68	neg	2	1	3
62	70	II	T2	N0	pos	pos	0	2.43	0.01	0.37	0.00	13.72	0.27	12.01	1.51	1.83	3.59	0.00	18.39	0.00	24.07	147.79	907.21	7.15	pos	0	0	1
63	53	III	T2	N1	pos	pos	0	11.06	0.00	7.78	0.00	49.36	0.01	64.78	1.95	12.28	1.62	0.00	58.39	0.00	115.40	554.31	10244.93	32.21	ND	1	1	2
64	47	III	T1		pos	pos	0	2.79	0.87	1.31	0.00	24.32	0.41	128.27	0.20	1.45	3.73	0.00	14.09	0.00	27.17	230.16	803.78	7.06	neg	0	0	1
65	43	II	T3	N1	pos	pos	0	3.09	0.58	0.26	0.00	11.19	0.18	23.56	0.00	1.19	3.73	0.00	10.93	0.00	26.40	49.11	291.04	6.70	neg	0	0	0
66	44	I	T1	N0	pos	pos	0	2.29	1.74	0.00	0.00	27.09	0.76	34.60	1.49	2.22	4.26	0.00	9.68	0.00	26.01	96.28	689.82	6.43	neg	2	1	2
67	54	II	T2	N0	pos	pos	0	2.13	0.01	0.78	0.00	12.92	0.81	26.19	0.43	2.73	4.06	0.00	21.10	0.00	29.50	43.24	365.06	7.42	neg	0	0	2
68	48	II	T2	N0	pos	pos	0	4.13	0.87	4.87	0.00	13.15	0.45	25.10	0.40	1.19	3.99	0.00	24.15	0.00	24.45	55.74	168.32	6.79	pos	1	0	1
69	64	III	T4	N1	pos	pos	0	5.22	1.45	0.99	0.00	25.70	1.35	136.43	0.00	1.07	4.20	0.00	27.54	0.00	25.62	147.28	193.00	6.52	pos	1	0	2
70	50	II	T3	N1	pos	pos	0	1.50	1.16	0.99	0.00	10.04	0.27	18.62	0.35	1.58	4.60	0.00	19.29	0.00	25.23	23.35	260.69	6.70	pos	1	1	1
71	57	II	T2	N0	pos	pos	ND	0.63	0.00	0.00	0.00	20.70	0.00	0.94	0.00	0.31	7.32	0.00	20.01	0.00	32.69	35.76	498.75	10.95	neg	0	0	0
72	62	II	T2	N1	pos	pos	0	5.11	8.38	13.36	0.00	95.63	2.42	404.97	0.87	6.56	394.07	5.16	22.91	0.00	1279.37	1543.66	962.55	120.82	pos	0	0	1
73	35	III	T1	N1	pos	pos	3+	2.76	0.87	5.29	0.00	14.41	0.94	90.65	0.23	2.34	3.99	0.00	10.93	0.00	24.84	68.74	994.80	6.88	pos	1	0	1
74	48	II	T2	N1	pos	pos	0	2.68	0.00	0.00	0.00	11.53	0.23	1.54	0.00	0.68	3.39	0.00	15.67	0.00	22.90	32.53	245.31	6.34	pos	1	1	1
75	71	II	T4	N1	pos	pos	0	4.73	10.98	30.23	0.00	65.22	3.05	364.53	0.55	7.20	343.08	2.57	18.50	0.00	87.37	1376.64	154.97	90.40	pos	0	0	1
76	71	I	Tx	N1	pos	pos	0	0.81	1.16	0.57	0.00	11.65	1.39	274.65	0.14	1.45	3.93	0.00	23.36	0.00	21.74	201.09	466.24	6.16	pos	0	0	2
77	50	I	T1	N1	pos	pos	0	0.68	0.87	0.00	0.00	9.23	0.76	25.81	0.08	0.68	3.53	0.00	9.35	0.00	20.96	102.66	202.63	5.89	pos	0	0	1
78	40	III	T1	N1	pos	pos	0	1.33	0.00	0.37	0.00	12.22	0.00	43.40	0.00	0.81	3.73	0.00	26.63	0.00	20.57	115.66	507.28	6.61	neg	0	0	1
79	38	III	T1	N1	pos	pos	0	0.62	4.63	1.94	0.00	8.77	1.48	4.69	0.26	3.37	6.41	0.00	16.13	0.00	27.56	24.37	159.14	7.15	pos	1	1	1
80	45	III	T2	N0	pos	pos	3+	2.87	1.16	0.57	0.00	29.85	0.76	67.35	1.16	0.94	3.86	0.00	15.45	0.00	26.40	122.80	582.93	6.88	pos	1	0	2
81	52	II	T2	N2	neg	pos	0	0.00	3.18	0.16	0.00	8.08	1.35	15.41	0.08	4.52	4.73	0.00	13.07	0.00	32.61	43.24	279.99	7.06	neg	0	0	1
82	78	I	T2	N0	pos	pos	0	0.00	0.01	0.36	0.00	11.19	0.00	9.07	0.00	0.00	5.25	0.00	13.41	0.00	30.64	25.28	451.42	9.15	neg	0	0	1
83	51	II	T3	N1	pos	neg	0	0.15	0.00	0.00	0.00	17.06	0.05	0.00	0.11	1.58	3.86	0.00	13.87	0.00	20.96	50.38	385.66	6.34	neg	0	0	1
84	60	III	T2	N1	pos	pos	0	1.33	0.00	6.13	0.00	9.46	0.00	0.32	0.00	17.17	4.73	0.13	11.04	0.00	15.52	83.28	446.86	6.88	neg	1	0	1
85	56	II			pos	pos	0	1.42	1.16	0.00	0.00	9.00	0.27	0.06	0.14	1.07	4.60	0.00	13.98	0.00	20.18	7.03	223.36	6.70	neg	0	0	0
86	89	II	T2	N0	pos	neg	0	1.58	2.32	0.00	0.00	29.16	1.57	24.59	0.20	1.58	4.40	0.00	35.45	0.00	24.45	40.95	266.85	6.16	neg	1	1	1
87	68	II	T2	N0	pos	pos	0	0.00	0.29	7.60	0.00	10.96	0.14	121.40	0.00	1.83	4.06	0.00	57.26	0.00	21.35	8.05	87.17	6.70	neg	0	0	1
88	73	I	T2	N0	pos	pos	0	0.00	0.00	3.40	0.00	10.61	0.32	27.67	0.00	1.19	3.39	0.00	8.55	0.00	19.79	1208.08	658.65	6.07	neg	0	0	1
89	45	III	T2	N0	pos	neg	0	0.21	0.00	2.88	0.00	12.34	1.17	31.14	0.17	0.68	4.46	0.00	9.91	0.00	44.26	80.22	582.72	8.68	neg	0	0	1
90	76	II	T2	N1	pos	pos	0	10.92	1.16	3.30	0.00	8.54	0.00	80.89	0.08	1.96	4.26	0.00	46.52	0.00	22.90	0.00	134.25	7.42	neg	0	0	2
91	64	II	T1	N0	pos	pos	0	0.87	0.29	0.00	0.00	7.96	1.79	143.23	0.17	0.55	2.99	0.00	23.36	0.00	17.85	191.65	647.43	7.15	neg	0	0	1
92	59	II	T2		pos	neg	0	3.28	0.29	9.48	0.00	70.75	0.58	653.75	0.70	3.11	4.06	0.00	24.37	0.00	25.23	135.30	745.27	6.70	neg	1	1	1
93	67	II	T1	N1	pos	pos	0	6.02	1.45	1.10	0.00	13.03	0.27	12.33	0.05	1.58	4.06	0.00	21.78	0.00	25.62	81.24	992.68	6.97	neg	1	0	2
94	71	II	T2	N1	pos	neg	0	0.00	0.29	1.20	0.00	9.92	0.23	27.99	0.05	2.47	4.13	0.00	20.42	0.00	24.84	17.74	147.02	6.97	neg	0	0	1
95	59	II	T2	N1	pos	pos	0	1.11	0.58	0.00	0.00	9.34	0.58	8.67	0.03	1.19	3.66	0.00	11.49	0.00	22.51	58.80	695.01	6.70	neg	0	0	1
96	55	II	T2	N0	pos	pos	0	2.59	4.05	0.00	0.00	12.11	0.00	11.11	0.11	0.43	17.80	0.00	76.47	0.00	88.54	48.60	224.91	11.74	pos	0	0	1
97	63	II	T1	N0	pos	neg	0	3.74	2.32	0.00	0.00	4.39	2.87	299.49	1.72	1.45	5.27	0.00	5.28	0.00	19.79	79.96	477.75	6.70	neg	1	1	2
98	69	III	T4	Nx	pos	pos	0	11.50	0.00	99.61	0.00	18.56	1.75	95.92	0.73	5.92	6.25	0.00	33.98	0.00	55.53	78.43	1120.18	9.94	neg	1	0	3
99	76	III	T4	N1	pos	pos	0	1.66	0.58	3.93	0.00	20.17	1.97	206.15	0.49	2.60	4.66	0.00	28.22	0.00	25.23	146.26	359.30	6.79	pos	0	0	1
100	69	II	T2	N0	pos	pos	0	0.32	0.00	0.00	0.00	19.71	0.00	68.05	0.20	2.73	4.06	0.00	13.98	0.00	17.07	146.77	272.28	5.80	pos	1	0	1
101	67	I	T1	N0	pos	pos	0	0.00	0.00	1.41	0.00	27.20	0.00	12.52	0.00	1.19	3.66	0.00	12.51	0.00	21.35	36.10	208.63	6.07	neg	0	0	1
102	72	I	T2	Nx	pos	neg	0	0.46	1.16	15.98	0.00	16.60	0.00	68.12	0.35	2.09	4.20	0.00	24.83	0.00	26.01	278.35	274.48	6.70	neg	2	1	1
103	73	III	T1	N0	pos	pos	0	0.26	0.00	1.10	0.00	10.73	0.00	51.87	0.05	1.45	3.86	0.00	21.89	0.00	22.90	39.67	318.30	6.34	neg	0	0	1
104	71	II	T2	N2	pos	pos	0	0.00	0.00	0.05	0.00	7.73	0.18	27.61	0.40	2.09	3.86	0.00	27.54	0.00	31.06	45.54	589.29	6.07	neg	2	1	3
105	83	II	T2	N1	pos	pos	0	0.00	1.45	0.00	0.00	14.53	0.85	48.66	0.52	2.47	3.73	0.00	5.73	0.00	19.79	29.98	303.86	6.07	neg	1	0	1
